# Volatile Metabolism of Wine Grape Trincadeira: Impact of Infection with *Botrytis cinerea*

**DOI:** 10.3390/plants11010141

**Published:** 2022-01-05

**Authors:** Helena Santos, Catarina Augusto, Pedro Reis, Cecília Rego, Ana Cristina Figueiredo, Ana Margarida Fortes

**Affiliations:** 1 BioISI—Biosystems and Integrative Sciences Institute, Faculty of Sciences, University of Lisbon, Campo Grande, 1749-016 Lisboa, Portugal; hfsantos@fc.ul.pt (H.S.); catarinasau@gmail.com (C.A.); 2 LEAF—Linking Landscape, Environment, Agriculture and Food-Research Center, Associated Laboratory TERRA, Instituto Superior de Agronomia, Universidade de Lisboa, Tapada da Ajuda, 1349-017 Lisboa, Portugal; pedroreis@isa.ulisboa.pt (P.R.); crego@isa.ulisboa.pt (C.R.); 3 Centro de Estudos do Ambiente e do Mar (CESAM Lisboa), Faculdade de Ciências da Universidade de Lisboa, Centro de Biotecnologia Vegetal (CBV), DBV, C2, Piso 1, Campo Grande, 1749-016 Lisboa, Portugal; acfigueiredo@fc.ul.pt

**Keywords:** *Vitis vinifera*, volatile organic compound, *Botrytis cinerea*, necrotrophic pathogen, plant defense, aroma

## Abstract

The aroma of grapes is cultivar dependent and is influenced by *terroir*, vineyard practices, and abiotic and biotic stresses. Trincadeira is a non-aromatic variety associated with low phenolic content and high sugar and organic acid levels. This cultivar, widely used in Portuguese wines, presents high susceptibility to *Botrytis cinerea*. This work aimed to characterise the volatile profile of Trincadeira grapes and how it changes under infection with *B. cinerea*. Thirty-six volatile organic compounds were identified, from different functional groups, namely alcohols, ester acetates, fatty acid esters, fatty acids, aldehydes, and products of the lipoxygenase pathway. Both free and glycosidic volatile organic compounds were analysed by Gas Chromatography and Gas Chromatography coupled to Mass Spectrometry for component quantification and identification, respectively. A multivariance analysis showed a clear discrimination between healthy and infected grapes with 2-*trans*-hexenal and isoamyl-acetate among the compounds identified as negative and positive markers of infection, respectively. Ester acetates such as 2-phenylethyl acetate, isoamyl acetate, and 2-methylbutyl acetate were present in higher contents in infected samples, whereas the contents of several fatty acid esters, such as ethyl decanoate and ethyl dodecanoate, decreased. These data were integrated with quantitative PCR data regarding genes involved in volatile metabolism and showed up-regulation of a gene coding for Hydroperoxide Lyase 2 in infected grapes. Altogether, these changes in volatile metabolism indicate an impact on the grape quality and may be related to defence against *B. cinerea*. The presence/absence of specific compounds might be used as infection biomarkers in the assessment of Trincadeira grapes’ quality.

## 1. Introduction

*Vitis vinifera* grape aroma may depend on many conditioning factors, including grape variety and its metabolism, soil, and climate. All of these factors influence the compounds present in the grapes and their concentrations, which contribute to the individual aromatic profile of wines [1]. *Vitis vinifera* cultivar (cv.) Trincadeira is a highly important Portuguese cultivar used in dry red blend wines from the Alentejo, Douro, and Dão regions, and it is considered a non-aromatic grape variety with potential for aromatic wines. It has an irregular yield depending on weather conditions and diseases, making its wine quality inconsistent throughout the years [2,3].

One of the main contributors to wine aroma are grape-derived aroma compounds such as volatile organic compounds (VOCs), which result from grape metabolism [4]. Volatile organic compounds include organic classes such as alcohols, aldehydes, esters, fatty acids, benzenoids, and others and contribute to aroma with different nuances, such as specific fruits, flowers, honey, grass, vinegar, and others. They can be found in their free or bound forms in different proportions, depending on if the cultivar is aromatic or non-aromatic [4,5]. In grapes, free form VOCs usually represent the smaller group of VOCs, being predominantly formed by endogenous enzymes when grapes are crushed [6]. In general, VOCs can be bound by a covalent link with compounds such as sugars or amino acids, limiting their chemical and sensory properties. The bound VOCs form a pool of aroma precursors and constitute the main potential aroma of grapes, as they do not contribute readily to the flavour. Glycoconjugated VOCs are one of the most common types. Glycosides are produced by glycosyl transferase enzymes that bound aglycones with single glycoside or disaccharide glycoside, the last being the most common in grapes, with a sugar linked to a glucose by a β-glycosidic linkage [5,6,7,8]. These aroma precursors can release the aglycone during yeast and malolactic fermentation, during exogenous glycosidase (GS) activity, and due to acid hydrolysis, making VOCs available in wine aroma [8,9,10].

Among the different VOCs classes, terpenes are the largest and include compounds such as the oxygen-containing terpenes geraniol or linalool. They are present in muscat-related grapes, which are highly aromatic cultivars. They can be present both in skin and pulp of berries, free and glycoconjugated [4,11,12]. Another family of VOCs is the 6-carbon (C6)-compounds, derived from the lipoxygenase (LOX) pathway. The precursors can be either linoleic acid or α-linolenic acid, and by the actionsof LOX, hydroperoxide lyase (HPL) and alcohol dehydrogenase (ADH), it can catalyse the production of either *cis*-3-hexenal or hexanal, and derivative products including *trans*-2-hexenal, *trans*-2-hexenol, *cis*-3-hexenol, and *n*-hexenol. This family of VOCs is associated with green notes aroma, which tend to decrease with ripening [11,13,14].

Present also in most fruits, the esters, derived from fatty acids, sugar, or amino acids, play an important role in wine aroma [8]. The most important esters in wine are the fatty acid ethyl esters and acetates, such as ethyl acetate, ethyl octanoate, ethyl decanoate, hexyl acetate, or isoamyl acetate. The aromas resemble that of fruits and flowers, among others [8,15]. Phenylpropanoids and benzenoid compounds derive from the aromatic amino acid phenylalanine. Their synthesis pathway involves many enzymes, such as *O*-methyltransferase (OMT), an enzyme that catalyses the methylation of the substrate [4,16,17]. In wine making, phenylalanine derivatives are metabolised by yeasts into compounds with floral aromas, such as 2-phenylethyl acetate [8]. Among the factors that might induce changes in quantity or quality of VOCs in plants are plant diseases, in particular fungal infections [18]. *Botrytis cinerea* is a necrotrophic pathogen that causes the grey mould disease, killing and destroying berries, leading to serious losses in many crop species [2,19]. Trincadeira is a cultivar highly prone to infection with *B. cinerea* starting from blooming to being fully ripe, leading to cell death and rotten berries [2]. The detection of *B. cinerea* in fields is fundamental for quality control and phytosanitary management of diverse crops. The main practices are based on visual inspection, which not only requires extensive knowledge but also might be subjective, as fruits can be asymptomatic due to latent infections [20,21]. Additionally, in big harvest batches, it might be difficult to identify infected fruits. As a first approach to this problem, VOCs can potentially be used as diagnostic markers of infection [22,23]. Several studies report on changes in VOCs in plant organs upon infection by *B. cinerea* [20,24,25]. Vandendriessche et al. [24] addressed these changes in strawberry, where the levels of aldehydes, lactones, and terpene alcohols decreased and the compounds 2-methyl-1-butanol, 2-methyl-1-propanol, 1-octen-3-ol, and ethanol increased in abundance with increasing infection rate. Jansen et al. [20] detected changes in the LOX pathway-derived products in tomato leaves upon infection, whereas Aprea et al. [25] observed changes in terpenes in different cultivars of raspberries upon infection by *B. cinerea*, which might be associated with resistance to the pathogen [25].

Changes in VOCs may occur due to environmental conditions, nutrient’s availability, and interactions with other plants or as an attempt to defend against pathogens by emitting toxic VOCs [26]. Many transcriptomic studies have unravelled the mechanisms underlying the aromatic pathways [11,16,27,28]. The integration of data on gene expression and VOCs profiles can elucidate not only the molecular basis of fruit aroma and quality but also defensive responses to *B. cinerea*.

Considering Trincadeira’s high susceptibility to *B. cinerea*, markers of infection can be of great use to guarantee the quality of grapes and wine. The main purpose of this study is therefore to study VOCs metabolism in Trincadeira grapes at the harvest stage and to identify changes under infection by *B. cinerea*.

## 2. Results

### 2.1. Phenotypic Characterization, Total Phenolic Content, and Anthocyanin Quantification in Infected and Mock-Treated Grape Berries

**Figure 1:**
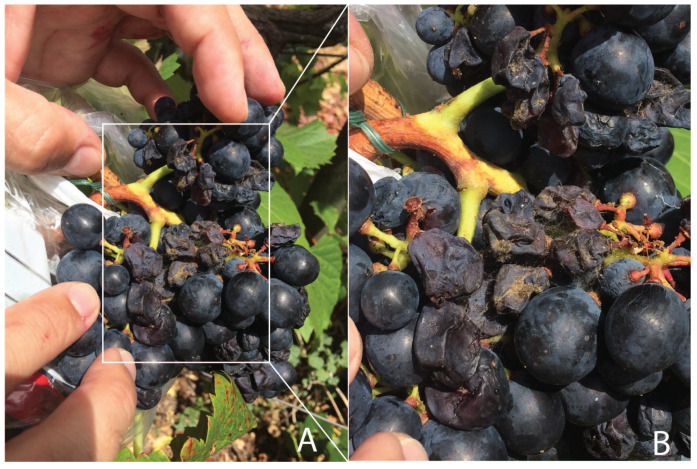
(**A**) Cluster of *Vitis vinifera* cv. Trincadeira infected with *Botrytis cinerea* at the harvest stage (EL38). Symptomatic infected grapes in the centre of the cluster show signs of dehydration, and sporulation of *B. cinerea* was visible, which is highlighted in (**B**).

Trincadeira clusters were infected at Eichhorn and Lorenz (EL)29 [29] as previously described [2] and were collected at EL38, corresponding to the harvest stage. The infected harvested clusters showed heavy symptoms of infection by *B. cinerea*, as shown in Figure 1. Sporulation was clearly noticed in the highly compact Trincadeira clusters.

**Figure 2:**
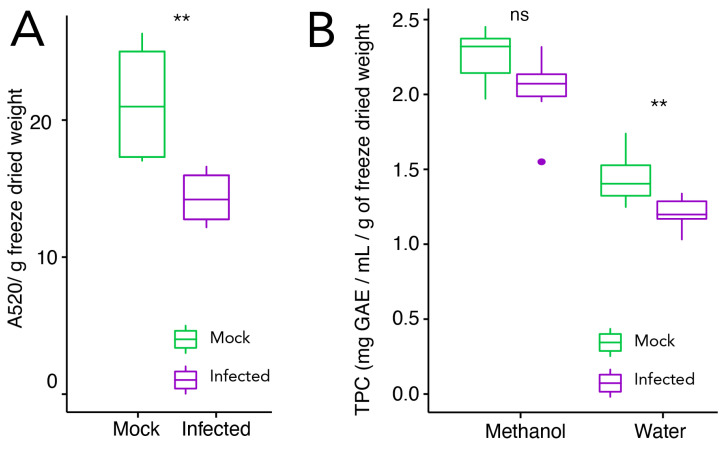
Evaluation of (**A**) total anthocyanins content (absorbance 520 nm $g^{-1}$ freeze dried weight) and (**B**) total phenolic content (TPC) extracted with methanol or water (gallic acid equivalent (GAE) mg m$L^{-1}$ $g^{-1}$ freeze dried weight) in *V. vinifera* cv. Trincadeira grapes at the harvest stage (EL38) (mock and *B. cinerea* infected). Box-and-whisker plots represent the minimum, first quartile, sample median, third quartile, maximum, and outliers. Total anthocyanins data were analysed with the Wilcoxon–Mann–Whitney test, and TPC data were analysed with the *t*-test. Asterisks indicate significant differences between control and infected samples within each method: ** *p* value < 0.01; ns: not significant.

As the content of secondary compounds such as phenols and anthocyanins indicate ripening stage and may play a role in plant defence against pathogens [30,31], both were extracted and quantified. Total phenolic content (TPC) extracted in methanol, a less hydrophilic solvent and therefore rich in compounds such as hydroxyphenylacetic acids or stilbenes, shows no changes upon infection, though a tendency for a potential decrease upon infection can be seen. The TPC extracted in water, a solvent that extracts mainly hydrophilic compounds such as hydroxybenzoic acids, anthocyanins, flavonoids, or tannins [13,32], is reduced upon infection (Figure 2B). The total anthocyanins content is lower in infected samples than in control samples (Figure 2A).

### 2.2. Profiling of Volatile Organic Compounds in Healthy and Infected Berries

A total of 36 different free and glycosidic VOCs were detected from either the control or infected samples using Gas Chromatography—Mass Spectrometry (GC–MS) and Gas Chromatography—Flame ionization detector (GC–FID). The relative amounts of the identified components after normalization are listed in Table 1, following their elution order on the DB-1 fused-silica column as described in the Materials and Methods section. The detected VOCs belong to the families of alcohols, fatty alcohols, aromatic alcohols, fatty acids, acids, alkanes, C6 derivatives from LOX pathway, ester acetates, and fatty acid esters. The full list can be found in Table S1.

Twenty-one free VOCs were detected in the samples, mainly represented by C6 alcohols and aldehydes, such as *n*-hexanol and 2-*trans*-hexenal. Infected grapes showed some degree of fermentation, with the higher relative amounts of ethanol and ethyl acetate. Twenty-eight VOCs were detected after the removal of compounds’ glycosidic moiety. Despite ethanol being the main component in both cases, there were some striking differences between control and infected grapes’ glycosidic bound VOCs. Apart from ethanol, the control grapes’ glycosidic bound VOCs were dominated by fatty acid derived esters such as ethyl decanoate and ethyl dodecanoate, whereas ethyl acetate was the second main component in infected grapes (Table 1 and Table S1).

**Figure 3:**
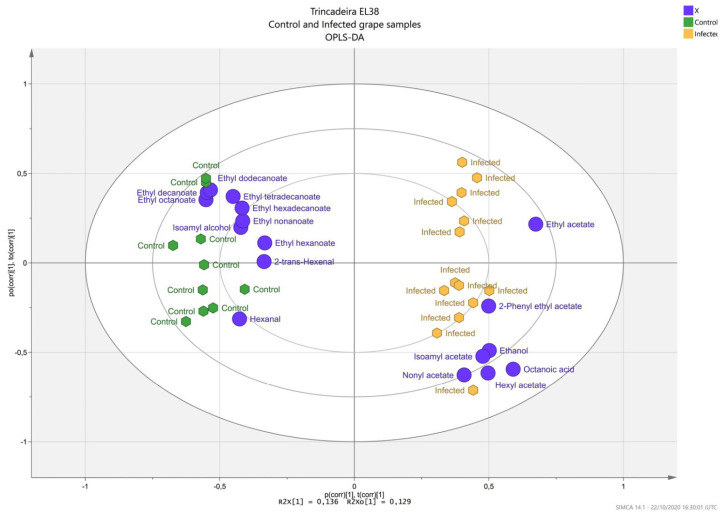
Biplot Orthogonal Projections to Latent Structures—Discriminant Analysis (OPLS–DA) of *V. vinifera* cv. Trincadeira samples upon infection by *B. cinerea*; infection status was selected as the Y-variable. The samples show a separation according to volatile organic compounds. Samples are coloured according to the following condition: green hexagons represent control samples (four biological and two–three technical replicates); yellow hexagons represent infected samples (seven biological and two technical replicates); purple circles represent the volatile compounds.

Principal component analysis (Figure S1) including both free and glycosidic VOCs provided a global overview, indicating a trend in separation between control and infected samples, with the first two principal component explaining 48% of the variance. Samples infected with *B. cinerea* were separated from the other samples along PC1. The resulting Orthogonal Projections to Latent Structures—Discriminant Analysis (OPLS–DA) (Figure 3) was used to model and identify the main contributors to the separation between mock and infected samples. Only the variables that had a variable influence on projection (VIP) score above 1 were considered for the biplot. Along the PC1, *B. cinerea* infected samples were separated from mock samples, and separation was driven by ester acetates (infected) and fatty acid esters (control). Further statistical differences in VOCs between the control and infected samples were confirmed by comparison tests. The VOCs detected but not quantified in both the control and infected samples were excluded from the analysis (trace VOCs were those considered < 0.05%).

**Figure 4:**
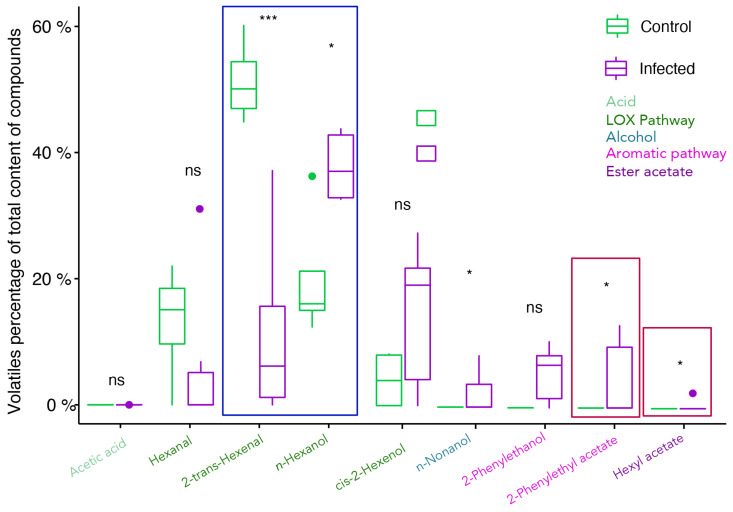
Evaluation of free volatile organic compounds in *V. vinifera* cv. Trincadeira grapes at the harvest stage (EL38) (mock and *B. cinerea* infected). Sample quantities were normalised for the percentage of total content of compounds (total compounds not shown). Box-and-whisker plots represent the minimum, first quartile, sample median, third quartile, maximum, and outliers. Data were analysed with the *t*-test or Wilcoxon–Mann–Whitney test depending on non-parametric/parametric distribution. Asterisks indicate significant differences between control and infected samples within each compound. * *p* value < 0.05; ** *p* value < 0.01, *** *p* value < 0.001; ns: not significant.

In the free fraction, five components, *trans*-2-hexenal, *n*-hexanol, *n*-nonanol, 2-phenylethyl acetate, and hexyl acetate, were statistically different upon infection (Figure 4). Regarding C6 VOCs from the LOX pathway, there was an increase in *n*-hexanol and a decrease in *trans*-2-hexenal. The fatty alcohol *n*-nonanol, 2-phenylethyl acetate, and hexyl acetate were only detected in infected samples.

Only small traces of acetic acid in the free fraction were detected. Acetic acid is usually a sign of decomposing state and fruit rotting [33]. Its absence reflects the edible ripe stage of the grapes. Acetic acid can also be a product of co-infection by acetic acid bacteria or sour rot [34], but its levels do not seem to reflect this opportunistic infection.

**Figure 5:**
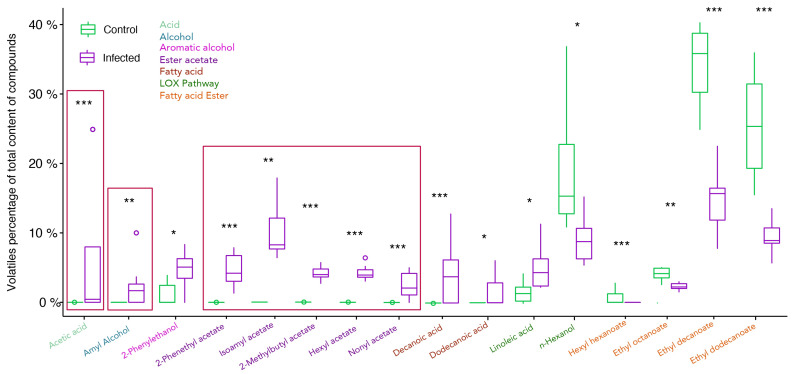
Evaluation of glycosidic volatile organic compounds of *V. vinifera* cv. Trincadeira grapes at the harvest stage (EL38) (mock and *B. cinerea* infected). Sample quantities were normalised for percentage of total content (total compounds not shown). Box-and-whisker plots represent the minimum, first quartile, sample median, third quartile, maximum, and outliers. Data were analysed with the *t*-test or Wilcoxon–Mann–Whitney test depending on non-parametric/parametric distribution. Asterisks indicate significant differences between control and infected samples within each compound. * *p* value < 0.05, ** *p* value < 0.01, *** *p* value < 0.001.

The direct analysis of the grape juice revealed a limited number of VOCs, confirming the non-aromatic grape profile. Therefore, grape juice from the same samples was treated with β-glycosidase to cleave VOCs glycosidic bonds. This revealed a high diversity of VOCs, showing the potential for aromatic wines from cv. Trincadeira grapes. In the VOCs obtained after glycosidase treatment, 22 out of the 28 glycosidic VOCs showed statistical differences upon infection (Figure 5). After the removal of glycosidic moiety, the VOCs hexanoic acid, octanoid acid, isopropyl alcohol and hexanal were detected only in infected grapes and only in trace amounts (<0.05%). Ethanol, ethyl acetate, and isopropyl alcohol were all statistically higher in infected samples but, due to their high percentage, were removed from the calculations, as explained in the Material and Methods section. Linoleic acid, a precursor from the LOX pathway increased in content upon infection and its derivative *n*-hexanol decreased in content. Five ester acetates, isoamyl acetate, 2-methyl butyl acetate, hexyl acetate, nonyl acetate, and 2-phenethyl acetate, were detected only in infected samples, representing 30% of total glycosidic VOCs. Decanoic acid and dodecanoic acid were also detected only in infected samples increasing from 1% to 11%. The aromatic alcohol 2-phenylethanol increased upon infection. Fatty acids esters decreased upon infection from 76% to 34%, namely hexyl hexanoate, ethyl octanoate, ethyl decanoate, and ethyl dodecanoate.

Opposite to the free VOCs fraction, acetic acid was present above trace amounts only in infected samples in the glycosidic fraction. It might have been released during the bond cleaving by β-glycosidase or may be related to the presence of laccases, which are oxidative enzymes produced by *B. cinerea* [33,35].

### 2.3. Expression of Genes Involved in Volatile Compound Metabolism

**Figure 6:**
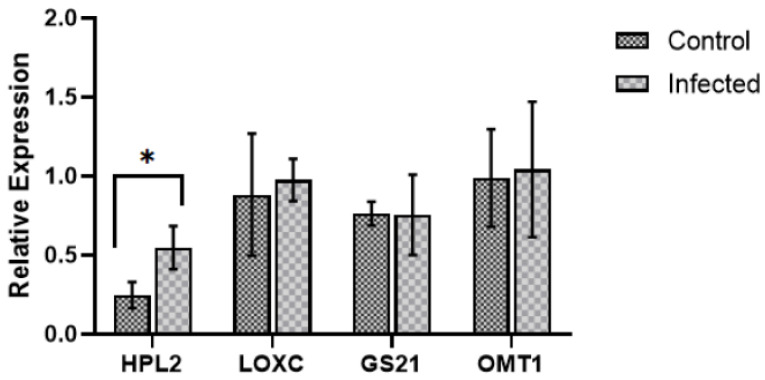
Expression of genes coding for hydroperoxide lyase (HPL)2, lipoxygenase (LOX)C, glycosidase (GS)21, and *O*-methyltransferase (OMT)1 in *V. vinifera* cv. Trincadeira grapes at the harvest stage EL38 (mock/control and *B. cinerea* infected). Four controls and four infected samples were analysed by qPCR. Barplot represent the relative expression means, and error bars are standard deviation. Data were analysed with the non-parametric Wilcoxon–Mann–Whitney test. Asterisks indicate significant differences in gene expression between control and infected samples within each method: * *p* value < 0.05.

In order to gather insights into molecular regulation of VOCs metabolism, a total of 16 genes involved in volatile metabolism were selected, and their expressions were assessed using quantitative polymerase chain reaction (qPCR). Three genes coding for HPL, three ADH, three lipoxygenase (LOX), five GS, and two OMT were selected based on Rambla et al. [36], who integrated gene expression with metabolic profiling in *V. vinifera* cv. Tempranillo and cv. Airén at different time points of development and ripening; the selected genes were chosen as relevant candidates for aroma development at the harvest stage. Gene expression is shown in Figure 6.

The first enzymatic step in the LOX pathway catalyses linoleic acid or α-linolenic acid in fatty acid hydroperoxides. We analysed three LOX genes, *VVLOXA*, *VVLOXC*, and *VVLOXD*, that were not differentially expressed (Figure S2). The resulting products were then metabolised by HPL, producing short-chain aldehydes that can be isomerised. Three HPL genes were tested: *VVHPL2*, *VVHPL5*, and *VVHPL6*. The gene *VVHPL2* showed a statistically significant increase in expression in infected samples (Figure 6). A third relevant enzyme family is the ADH, which oxidases C6-alcohols to the corresponding aldehydes. The genes *VVADH1*, *VVADH2*, and *VVADH3* were analysed; however, no change was noticed in their expression under infection (Figure S2). As most of detected VOCs were present in the glycosidic form, five genes coding for GS were also investigated: *VVGS6*, *VVGS9*, *VVGS16*, *VVGS21*, and *VVGS25*, but none were differentially expressed under infection. The same holds true for *VVOMT1* and *VVOMT2*, genes coding for enzymes that play an important role in aroma-related pathways [37].

## 3. Discussion

Volatile organic compounds are involved in plant metabolic pathways essential for development, reproduction, and defence [38]. Stresses can lead to qualitative and quantitative VOCs changes. Upon fungal infection, VOCs might act as toxins or defensive compounds or as pathogens’ sources of energy and infection potentiators. The necrotrophic fungus *B. cinerea* leads to fruit rotting or the bunch rot disease, resulting in severe production losses. However, in *V. vinifera* grapes, depending on the extension and conditions of the infection, it might cause noble rot, a biological process that leads to exquisite and sweet wines [39].

Understanding how fruits respond to this necrotrophic fungus is essential for grapevine improvement and wine production sustainability. Many studies have characterised the aroma of different grape cultivars, in both healthy and infected grapes, unravelling diverse VOCs associated with *B. cinerea* infection [2,11], but no markers of infection have clearly been identified as transversal to all cultivars upon this disease. Gas Chromatography Headspace Solid Phase Microextraction (HP–SPME) technology allows for the detection of grape VOC composition, and the comparison between infected and healthy grapes for VOCs markers of infection. Studies using glycosidases contribute to the understanding of the glycosidic bound VOCs fraction, which is of high relevance in wine making, especially in non-aromatic cultivars such as Trincadeira.

### 3.1. The C6-Volatile Compounds Are Altered in Both Free and Glycosidic Fractions

The lipoxygenase pathway can use both linoleic acid and α-linolenic acid precursors, leading to green leaf aroma C6 VOCs such as hexanal and *n*-hexanol, and *trans*-2-hexenal and *trans*-2-hexenol, respectively. The percentage of the C6 VOCs decreases upon infection in both free and glycosilated fractions, from 90% to 71% and from 20% to 14%, respectively. Interestingly, there is an increase in free *n*-hexanol upon infection with *B. cinerea* and a decrease in free *trans*-2-hexenal. These changes in C6 VOCs most likely affect the green notes perception in the aroma of the grapes. The LOX pathway can be induced by a variety of biotic stresses such as fungal pathogen infection [40] and the resulting products—C6, C9, and jasmonic acid (JA)—serving as signals of stress [4]. Some C6 VOCs, including *n*-hexanol, have been associated with antifungal activity and can inhibit *B. cinerea* growth [4,41,42,43]. Infection experiments conducted by Schueuermann et al. [44] in *V. vinifera* cv. Chardonnay grapes showed that, in two out of three vintages, *B. cinerea* infected samples had a reduced concentration of *trans*-2-hexenal. On the other hand, Tosi et al. [45] detected a reduction in the concentration of *n*-hexanol in the noble rot botrytised *V. vinifera* cv. Garganega grapes in contrast with our results in grey mould disease, suggesting that this compound may be a marker of infection to grey mould and not noble rot and is associated with low quality. Considering that ADH is not differentially expressed in infected samples, the free *n*-hexanol may come from the inhibition/degradation of ADH that converts *n*-hexanol in hexanal or de novo synthesis [13].

Previously, levels of *trans*-2-hexenal were associated with anthocyanins content under infection [13]; in our study, both *trans*-2-hexenal and anthocyanins are reduced in Trincadeira-infected grapes. Both compounds, when present in specific quantities, may reduce susceptibility to *B. cinerea* [30,46]. It has also been described that *B. cinerea* infection leads to degradation of anthocyanins, usually through laccases secretion [31,33,47]. The *trans*-2-Hexenal as also been associated with defence by inducing the expression of genes involved in resistance to *B. cinerea* [48]. However, at low concentrations, *trans*-2-hexenal may promote hyphal growth [49].

Jasmonic acid is a LOX pathway-derived hormone well-known as playing a major role in biotic stress defence. Previous hormonal profiling showed increased content in jasmonate–isoleucine, a compound with strong signalling properties, in infected Trincadeira grapes at the harvest stage [50]. Jasmonate-mediated signalling, also previously shown to be activated in these samples [50], is known to trigger back C6 VOCs emission in a positive feedback system [32]. Alternatively, *B. cinerea* might manipulate C6 VOCs levels for a less effective defence but still attracts herbivores that serve as vectors for conidia dispersion [13,51].

Regarding gene expression analysis, qPCR data only showed an increase in HPL2 mRNA in infected grapes. This can be due to an earlier transcriptional reprogramming, considering that previous RNAseq data from infected green grapes collected in the same vineyard [2] showed an upregulation of these genes upon infection. Alternatively, other genes from the gene families associated with volatile metabolism are up-regulated in Trincadeira, compared with the cultivars studied by Rambla et al. [36]. The enzyme HPL is involved in the LOX pathway and converts hydroperoxides in aldehydes. Previous in vivo studies confirmed that VvHPL2 presents function as 9/13HPL that catalyses the formation of both C6 and C9 aldehydes [52]. Although HPL2 catalyses 3-*cis*-hexenal production, only the isomer 2-*trans*-hexenal was detected. This can be due to spontaneous isomerization or due to high 3-*cis*:2-*trans*-enal isomerase activity, which is hypothesised to be higher in ripe berries [52] but generally reduced in fungal infections [51]. No traces of C9 aldehydes were detected, which may be due to lack of strong 9-LOX activity to catalyse the substrate or a higher affinity by HPL2 to 13-hydroperoxydes [52]. Shiojiri et al. [42] showed that the overexpression of HPL in *Arabidopsis thaliana* resulted in higher resistance of the transgenic plants against *B. cinerea*, most likely due to increased concentrations of C6 VOCs emitted by plants upon infection, reflecting the putative role of VOCs’ metabolism in grape defensive mechanisms. The literature also describes that membrane damage by *B. cinerea* effector lipases may release fatty acid substrates that become available for the LOX pathway and C6 VOCs production [51]. This agrees with the fact that C6 VOCs are one of the first class of VOCs being released upon biotic stress and when grapes are crushed [13,32,51].

### 3.2. Alcohols Increase upon Infection, Contributing to Specific Grape Aroma

Alcohols changed upon infection both in free and glycosidic fractions. Alcohols have physical–chemical properties that can induce membrane disruption and interfere with cell metabolic functions [53,54]. The alcohols *n*-nonanol, with floral/fruity aroma [55,56], and isoamyl alcohol, with fusel aroma [57], were increased in the free fraction. Isoamyl alcohol is known for its anti-fungal properties [53], and its levels are usually associated with activity of *B. cinerea* laccases [33].

In the glycosidic fraction, ethanol levels were higher in infected samples. These results might be due to a product of spontaneous fermentation, as there are higher amounts of yeasts in the microbiome of infected grapes compared with healthy grapes [58]. Interestingly, ethanol is considered as having inhibitory effect in *B. cinerea* colonies [53]. Another alcohol statistically relevant in this fraction is amyl alcohol, with a fusel-like odour [59], that was only detected in infected samples of the glycosidic fraction, usually relevant in plant metabolism to form esters. The alcohol 2-phenylethanol is characterised by its pleasant rose-like odour and increases in the glycosidic fraction of infected samples. Changes in this alcohol have been described in other cultivars, increasing upon infection by *B. cinerea* [33,44], and in botrytised wines [60]; however, the opposite was also reported in other studies, with its decrease upon infection by *B. cinerea* [45,61]. The role of 2-phenylethanol in defence and antifungal properties has been described in many pathogens [62], with a reduced toxicity when glycosylated [63]. Both Yalage Don et al. [53] and Liu et al. [64] showed that 2-phenylethanol significantly suppressed *in vitro B. cinerea* and *Alternaria alternata* colonies growth. Parafati et al. [65] detected an effect of 2-phenylethanol in spore germination, growth, and toxin production of *Aspergillus flavus*. The presence of this compound in these samples might be due to its role in plant defence against *B. cinerea*.

In general, the changes in the alcohols most likely contribute to a grape rotten aroma due to the fusel-like alcohols, even though 2-phenylethanol is mostly reported as contributing to a pleasant aroma.

### 3.3. Ester Acetates Are Only Present in Infected Samples and Fatty Acid Esters Decrease under Infection

Ester acetates exhibited traces in control grapes and therefore were only quantified in infected grapes. They were mostly present in their glycosidic form, contributing to 27% of total VOC in this fraction. Nevertheless, the compounds 2-phenethyl acetate, hexyl acetate, and ethyl acetate were also quantified in the infected free fraction, representing only 5% of total VOCs. The ester acetates detected in the glycosidic fraction in infected samples include 2-phenethyl acetate, with a rose aroma [57]; hexyl acetate, with a sweet fruit aroma [6]; isoamyl acetate, with a banana aroma [57]; 2-methylbutyl acetate, with a floral/fruity aroma; and nonyl acetate, which has a mushroom-gardenia aroma [59]. Hexyl acetate is not usually present in grape juice [66] but in wine instead, deriving from C6 VOCs such as *trans*-2-hexenal, hexanal, *trans*-2-hexen-1-ol, and *n*-hexanol [66,67]. Isoamyl acetate is a key component in Pinotage wine [68] but not grapes. Interestingly, Filonow [69] reported that four acetate esters including ethyl acetate, hexyl acetate, and 2-methylbutyl acetate, stimulate conidial adhesion of different *B. cinerea* strains to the fruit skin prior to germination. Thus, these ester acetates found only in infected grapes might facilitate fungal colonization; their synthesis may involve alcohol acetyltransferases from *B. cinerea* since activity of these *V. vinifera* enzymes was only proven in vitro [67,70]. Regarding the free ester acetates, there is ethyl acetate, of which the aroma can either be fruity when diluted [71] or resembles nail polish remover [15], contributing more acetic acid to the vinegary aroma [72]. Its presence is common in rotten berries, and it belongs to the group of undesirable metabolites [73]. However, it does not appear to be specific for *B. cinerea* since other diseases can lead to its production [73], and may also be cultivar-dependent since Schueuermann et al. [44] did not detect it in cv. Chardonnay.

The VOCs that mainly constituted the glycosidic fraction in control samples were fatty acid esters (76% of total VOCs). These compounds have odours of wax/honey or fruit/floral and are considered important contributors to young wine aroma [1,74]. Upon infection, there was a decrease in several fatty acid esters such as ethyl octanoate, ethyl decanoate, ethyl dodecanoate, and hexyl hexanoate. This class of compounds accounted for 34% of total VOCs in the infected glycosidic fraction. Hexyl hexanoate is a product of the reaction between the alcohol *n*-hexanol and the acid hexanoic acid [75]. Considering that hexanoic acid was detected in low percentages in infected samples in the glycosidic fraction, and it is a precursor of *n*-hexanol and hexyl acetate as well, it is possible that, during infection, the synthesis of *n*-hexanol and hexyl acetate is favoured instead of hexyl hexanoate [51].

Ethyl dodecanoate decreased upon infection, while its precursor dodecanoic acid increased in infected samples. The same holds true for ethyl decanoate and its precursor decanoic acid. The decrease in fatty acid esters in wines from *B. cinerea* infected berries was reported by Tosi et al. [45]. Calvo-Garrido et al. [76] found that conidial germination and germ tube morphology of *B. cinerea* in *V. vinifera* berries were debilitated in the presence of the medium chain fatty acid dodecanoic acid. All of the detected fatty acids in our study are in fact medium-chain fatty acids and are only quantified in infected samples, contributing to 6% of glycosidic VOC. Medium-chain fatty acids may have antimicrobial activity and play an important role in defence and signalling [2,77]. We hypothesise that there is an attempt to increase fatty acids levels in detriment of the corresponding esters, so signalling cascades associated with defence can be initiated or structural defences can be established, as to our knowledge, fatty acid esters have not yet been described in the literature as having an apparent role in defence.

### 3.4. Volatile Organic Compounds Blend for a Global Aroma

The study of volatile metabolism in Trincadeira grapes under infection with *B. cinerea* clearly indicates that a defensive response is activated and that infection has a major impact in grapes´ aroma. Interestingly, *B. cinerea* can either develop the disease bunch root or lead to exquisite noble root wines. Dankó et al. [39] studied how cv. Furmint aroma changes in both circumstances. They detected higher quantity of 2-phenylethanol in bunch root compared with noble root. Other studies detected that noble rot botrytised wines have more VOCs than the corresponding healthy grapes, such as esters, fatty acids, lactones, furans, phenolic acid derivatives, thiols, and phenols [78].

Some off-flavours commonly found in grapes infected by *B. cinerea*, such as fenchol, geosmin, 1-octen-3-ol, or lactones, with mushroom or earthy odours [47,58,79] were not detected in this study. This fact highlights how different cultivars and *terroir* might influence the impact of *B. cinerea* infection. However, it is important to note that analytical methods may be different among studies, affecting direct comparisons. Strikingly, many compounds detected, such as esters acetate, are usually associated with de novo synthesis by yeasts during wine fermentation [1,80]. It would be interesting to further explore if *B. cinerea* activates the enzymatic machinery to synthesise these compounds or alternatively if it is dependent on the grape microbiome.

**Figure 7:**
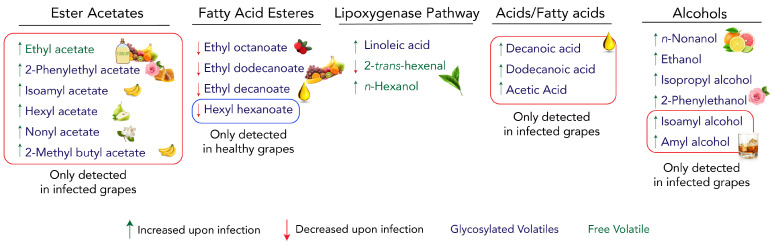
Main changes in volatile organic compounds (VOCs) in Trincadeira grapes upon infection by *Botrytis cinerea* and their respective type of aroma contribution. VOCs are separated by their organic family. Green upward-facing arrows represent an increase in the VOCs relative quantity, red downward-facing arrows represent a decrease in the VOCs relative quantity, red boxes highlight VOCs that were only detected in infected samples, and blue box highlights VOCs that were only detected in healthy samples. Names written in green change in the free fraction, and names written in blue change in the glycosidic fraction.

According to what has been documented for specific volatile compounds, the aroma of infected Trincadeira grapes may be characterised by a shift in the fruity aroma profile as well as less green and waxy properties. However, the high levels of ethyl acetate, isoamyl alcohol, acetic acid, and ethanol most likely mask them and overtake the aroma with a pungent unpleasant aroma (Figure 7). In addition, the reduction in anthocyanins and TPC in grapes probably lead to a wine with reduced astringency and colour [81].

Finally, our results highlight several VOCs, namely ester acetates, hexyl hexanoate, decanoic acid, dodecanoic acid, isoamyl alcohol, amyl alcohol, and 2-*trans*-hexenal, that may be used as putative biomarkers for *B. cinerea* presence in Trincadeira grapes. These biomarkers may be validated in other cultivars and/or tested for specificity compared with other fungal infections. This raises the opportunity to investigate how volatile metabolism is involved in defensive mechanisms of *V. vinifera*.

## 4. Materials and Methods

### 4.1. Fungal Infection of Berries, Sample Collection, and Processing

*Vitis vinifera* berry clusters were infected with *B. cinerea* in early June of 2018 at an experimental vineyard in Instituto Superior de Agronomia, University of Lisbon, Portugal. The *B. cinerea* isolate was collected from diseased grapevine plants and maintained in potato dextrose agar (Difco, Detroit, MI, USA), at 5 ${}^{\circ }C$. Conidia production was obtained by exposing inoculated Petri dishes with potato dextrose agar to continuous fluorescent light at 24 ${}^{\circ }C$. Conidia were harvested from 14- to 20-day-old cultures and collected by rubbing with phosphate buffer (0.03 M KH_2_PO_4_). A suspension of $10^{5}$ conidia m$L^{-1}$ was sprayed over the berry clusters at the developmental stage of peppercorn size (according to the modified EL system [29], stage EL29) following the procedure by Agudelo-Romero et al. [2]. Mock clusters were sprayed with phosphate buffer. Four mock and seven infected *V. vinifera* cv. Trincadeira bunches were collected at the harvest stage (EL38) in September 2018. Samples were kept for 24 h at 4 ${}^{\circ }C$ to mimic field practices. Fruits were frozen in liquid nitrogen and the seeds were removed and macerated to a thin powder for RNA extraction, the fruits were processed for VOCs quantification, or the fruits were freeze-dried for 96 h at 40 ${}^{\circ }C$ for anthocyanins and TPC quantification. For metabolic profiling, biological replicates were analysed independently, while for qPCR, some biological replicates were pooled to yield four independent biological replicates.

### 4.2. Isolation of Free and Glycosidic Bound Volatile Organic Compounds

Free and glycosidic bound VOCs were analysed by HP–SPME from the juice of freshly crushed non-infected (control) and infected Trincadeira grapes (approximately 100 g of grapes). For the collection of volatiles, the filtered juice (7 mL) was inserted into 22 mL clear vials, each sealed with a screw top solid cap containing a polytetrafluoroethylene (PTFE) liner. The vials were accommodated in a support rack inserted into a Bains Marie bath (Univeba).

Headspace VOCs were collected by HP–SPME using 100 μM polydimethylsiloxane (PDMS)-coated fibers (Supelco, Bellefonte, PA, USA) inserted into manually operated HP–SPME holders. Each holder needle was inserted through the PTFE liner of the vial, and VOCs sampling was carried out by coated fibre exposure for 30 min at 50 °C. Each HP–SPME fibre was thermally conditioned for up to 25 min at 250 ${}^{\circ }C$ before use, according to the manufacturer recommendations. Blank experiments using fibres in empty vials were carried out regularly. Four mock and seven infected biological samples were analysed. Juice from each sample was obtained twice for the free VOCs and the glycosidic bound VOCs analysis.

After isolation of the free volatiles, β-glycosidase from almonds (lyophilised powder, ≥ 2 units m$g^{-1}$ solid–Sigma-Aldrich®, St. Louis, MO, USA) (10 mg/L) was added to the remaining juice, according to the Svendsen and Merkx method [82]. The vials were kept in an orbital shaking incubator at 15 rpm, and hydrolysis was run for 24 h at 37 ${}^{-}C$. The volatiles obtained after hydrolysis were isolated by SPME as detailed above, with a fibre exposure time of 1 h at 50 ${}^{\circ }C$.

### 4.3. Headspace HP–SPME Chemical Analysis

Non-infected (control) and infected juice volatiles, and glycosidic bound volatiles isolated by HP–SPME were analysed by GC–MS for component identification and by GC for component quantification.

Gas Chromatography. Immediately after sampling, the HP–SPME needle was introduced into the split/splitless injector of a Clarus 400 gas chromatograph equipped with two flame ionization detectors, with a data handling system and a vaporising injector port into which two columns of different polarities were inserted: a DB-1 fused-silica column (100% Dimethylpolysiloxane, 30 m × 0.25 mm i.d., film thickness 0.25 μm; J & W Scientific Inc., Orangevale, CA, USA) and a DB 17HT fused-silica column [(50%-Phenyl)-methylpolysiloxane, 30 m × 0.25 mm i.d., film thickness 0.15 μm; J & W Scientific Inc., Orangevale, CA, USA]. The oven temperature was programmed from 45 ${}^{\circ }C$ to 175 ${}^{\circ }C$, at 3 ${}^{\circ }C$ $\min^{-1}$, then up to 300 ${}^{\circ }C$ at 15 ${}^{\circ }C$ $\min^{-1}$, and finally held isothermal for 10 min, for a total run time of 61.67 min. The analyte desorption was achieved in splitless mode for 1 min, and the gas chromatographic settings were as follows: injector and detector temperatures were 250 ${}^{\circ }C$ and 290 ${}^{\circ }C$, respectively; carrier gas was H_2_ at 30 cm $s^{-1}$. The percentage composition of the VOCs was computed by the normalization method from the GC peak areas without the use of correction factors in accordance with ISO 7609. The values shown represent the mean value of at least two injections per sample.

Gas Chromatography–Mass Spectrometry. Directly after sampling, the HP–SPME needle was introduced into the split/splitless injector of the GC–MS unit that consisted of a Perkin Elmer Clarus 600 gas chromatograph equipped with DB-1 fused-silica column (100% Dimethylpolysiloxane, 30 m × 0.25 mm i.d., film thickness 0.25 μm; J & W Scientific Inc., Orangevale, CA, USA) interfaced with a Perkin-Elmer Clarus 600T mass spectrometer (software version 5.4.2.1617, Perkin Elmer, Shelton, CT, USA). Injector and oven temperatures were as above: transfer line temperature, 280 ${}^{\circ }C$; ion source temperature, 220 ${}^{\circ }C$; carrier gas, helium, adjusted to a linear velocity of 30 cm $s^{-1}$; analytes desorption was achieved in splitless mode for 1 min; ionization energy, 70 eV; scan range, 40–300 u; and scan time, 1 s. The identity of the components was assigned by comparing their retention index (RI) to C4–C22 *n*-alkane indices, and the GC–MS spectra were from a laboratory-made library built based upon the analyses of reference essential oils, laboratory-synthesised components, and commercially available standards.

The volatile organic compounds levels were normalised as a percentage of the total VOCs content. As ethanol and others were in much higher percentage (Table S1) compared with the other VOCs, the low percentage VOCs changes and percentages were masked. Therefore, five compounds were suppressed from the total VOCs after an initial statistical analysis and the data was normalised for the remaining VOCs (Table 1).

### 4.4. Determination of Total Phenolic Content

Total phenolic content was determined by spectrophotometry using gallic acid as a standard in a modified method of Singleton and Rossi [83]. Four biological and four technical replicates were used in mock, and seven biological and one technical replicate were used in infected samples. Briefly, to 25 mg of the freeze-dried sample, 0.2 mL of distilled water was added. The samples were centrifuged at 12,900 rpm for 40 min and 1 mL of a 1:10 dilution of Folin–Ciocalteu’s reagent–water was added to the supernatant, which reacted for 10 min. Then, 0.8 mL of Na_2_CO_3_ (7.5% *w*/*v*) was added. The tubes were allowed to stand at room temperature for 30 min. In parallel, the same procedure was performed using methanol instead of water as initial solvent.

Absorbance was measured at 743 nm for water-based and 765 nm for methanol-based extraction. Total phenolic content was expressed as mg m$L^{-1}$ of GAE per mg of the lyophilised sample, derived from a calibration curve of gallic acid.

### 4.5. Determination of Total Anthocyanin Content

Anthocyanin concentration was measured as described previously [50]. To 50 mg of freeze-dried sample, 1.5 mL of trifluoroacetic acid (TFA)/methanol/water (0.05/80/20, *v*/*v*/*v*) was added. The reaction occurred for 1 h on ice. Samples were centrifuged at 13,000 rpm, 4 ${}^{\circ }C$ for 30 min. 100 μL of the supernatant was diluted in 1 mL of the extraction solution and let rest for 5 min. Total relative anthocyanin concentration was expressed as the absorbance value at 520 nm $g^{-1}$ of freeze-dried weight.

### 4.6. RNA Extraction and Purification for Transcriptional Profiling

RNA extraction was carried out according to Fortes et al. [84] with modifications by Coelho et al. [50]. Infected samples were pooled to obtain three independent biological replicates. Briefly, to the frozen thin powdered sample, an RNA extraction buffer (Tris-HCl 1 M pH 9, 1% SDS, 0.8% PVP-40, 0.5% β-mercaptoethanol) was added. The samples were extracted in chloroform/isoamylalcohol (24:1, *v*/*v*). To the supernatant, KCl 2 M was added. It was precipitated using 1/10 vol. CH_3_COONa 3 M and 0.8 vol. of isopropanol and, after, with LiCl 4 M, followed by washes with ethanol and solubilization in water. DNAse treatment and RNA purification were performed according to the suppliers’ instructions—Spectrum™ Plant Total RNA Kit (Sigma-Aldrich). The purity of RNA was confirmed using the Nanodrop ND-1000 spectrophotometer at the absorbance ratios of 260/280 nm and 260/230 nm. RNA integrity was confirmed in agarose gel.

### 4.7. Quantitative RT-PCR for Genes Involved in Volatile Metabolism

First-strand cDNA was synthesised from 2 μg of total RNA according to Fortes et al. [84]. Based on the VOCs alterations from the GC results, the genes considered relevant by Rambla et al. [36] involved in the pathways of those VOCs were selected and the corresponding primers were used (Table S2).

Quantitative PCR (qPCR) reactions were prepared using Thermo Scientific™ Maxima SYBR Green qPCR Master Mix and performed using the StepOne™ qPCR System (Applied Biosystems, Foster City, CA, USA). Cycling conditions were 95 ${}^{\circ }C$, 10 min; 42 cycles of 95 ${}^{\circ }C$, 15 s, 58 ${}^{\circ }C$ or 60 ${}^{\circ }C$, 40 s. Four control samples as well as four infected samples resulting from pools of groups of two biological samples during RNA extraction were analysed. Their expressions were determined using a standard curve, built with a serial dilution of mixture of all analysed samples cDNA, 1:1, 1:4, 1:16, 1:64, and 1:256 in triplicate and a negative control. The data were normalised using the expression curves of the actin gene (VIT04s0044g00580) and Elongation Factor 1 α gene (VIT06s0004g03220), which were the most stable according to the NormFinder software [2].

### 4.8. Statistical Analysis

Statistical data analysis was performed using R studio version 4.0.2 (22 June 2020). The minimum statistically significance was *p* value ≤ 0.05. Multivariance analysis with PCA and OPLS–DA were executed in SIMCA version 14.1.

## Figures and Tables

**Table 1:** Free and glycosidic bound volatile organic compounds expressed as a percentage of total VOC content, in both control and *B. cinerea* infected Trincadeira grapes. Compounds with relatively high percentages (ethanol, isopropyl alcohol, acetaldehyde, ethyl acetate, and isoamyl alcohol) were removed as described in the Materials and Methods section (full list of compounds available in Table S1). Free and glycosidic form VOCs were analysed in separate runs.

VOCs	RI	Free Form Percentage		Glycosidic Bound Percentage	
		Control	Infected	Control	Infected
Acetic acid	606	t	t	t	6.0
Amyl alcohol	839				2.9
Hexanal	840	13.0	7.6		t
2-*trans*-Hexenal	866	51.3	11.3		
*cis*-2-Hexen-1-ol	882	4.0	14.6		
*n*-Hexanol	882	21.1	37.9	19.3	9.3
Isoamyl acetate	882				10.4
2-Methyl butyl acetate	882		t	t	5.2
Hexanoic acid	970		t		0.2
Ethyl hexanoate	965			1.6	0.7
Hexyl acetate	995		0.5	t	4.3
2-Phenylethanol	1064	t	5.4	1.2	4.7
*n*-Nonanol	1148		2.3	1.8	4.1
Octanoic acid	1149				t
Ethyl octanoate	1177		0.6	4.8	2.3
2-Phenylethyl acetate	1222		4.3	t	4.7
Ethyl nonanoate	1273			2.6	0.3
Nonyl acetate	1300			t	2.4
Decanoic acid	1356			t	4.6
Hexyl hexanoate	1375			0.8	
Ethyl decanoate	1387			34.3	14.7
Dodecanoic Acid	1550				1.7
Ethyl dodecanoate	1580			25.5	9.4
Ethyl tetradecanoate	1774			2.1	1.0
*n*-Octadecane (C18)	1800	1.7	5.6		
*n*-Nonadecane (C19)	1900	6.1	2.3		
Ethyl hexadecanoate	1936			4.3	5.9
*n*-Eicosane (C20)	2000	2.7	4.3		
*n*-Heneicosane (C21)	2100	t	3.3		
Linoleic acid	2137			1.6	5.2
*n*-Docosane (C22)	2200	t	t		

RI: Retention index relative to C4–C22 *n*-alkanes on the DB-1 column. t: traces (<0.05%).

## Data Availability

Not applicable.

## Supplementary Materials

The following are available online at https://www.mdpi.com/article/10.3390/plants11010141/s1. Figure S1: Principal component analysis of total volatile organic compounds, explaining 48% of the variability. Green circles represent healthy samples, and yellow circles represent infected samples. A separation across the first principal component is clear; Figure S2: Plot with 2^−∆∆C_*T*_^ values between control and infected samples for all of the genes tested. Genes with value below 1 were not considered for the further experiments. The expression of certain genes (Figure 6) was further determined using a standard curve, as described in the Materials and Methods section; Table S1: Free and glycosidic bound volatile organic compounds expressed as percentage of total volatile organic compounds content in both control and *Botrytis cinerea* infected Trincadeira grapes. Free and glycosidic form volatile organic compounds were analysed in separate runs; Table S2: List of primers selected from the literature that were used for the qPCR analysis. Tm: Melting temperature in °C.
