# Supplementary material for: Volatile Metabolism of Wine Grape Trincadeira: Impact of Infection with Botrytis cinerea
Source: Plants (Basel). 2022 Jan 5;11(1):141. doi: 10.3390/plants11010141 (PMC8747702; doi:10.3390/plants11010141)
Supplement: Supplementary file 1 [file plants-11-00141-s001.zip › plants-1534493-supplementary.pdf]

Volatile metabolism of wine grape Trincadeira: impact of infection with *Botrytis cinerea*

Helena Santos[1], Catarina Augusto[1], Pedro Reis[2], Cecília Rego[2], Ana Cristina Figueiredo[3], and Ana Margarida Fortes[1]

[1]*BioISI – Biosystems and Integrative Sciences Institute, Faculty of Sciences, University of Lisbon, Campo Grande, 1749-016 Lisboa, Portugal*

[2]*Instituto Superior de Agronomia, Universidade de Lisboa, Tapada da Ajuda, 1349-017 Lisboa, Portugal*

[3]*Centro de Estudos do Ambiente e do Mar (CESAM Lisboa), Faculdade de Ciências da Universidade de Lisboa, Centro de Biotecnologia Vegetal (CBV), DBV, C2, Piso 1, Campo Grande, 1749-016 Lisboa, Portugal*

Supplementary Data

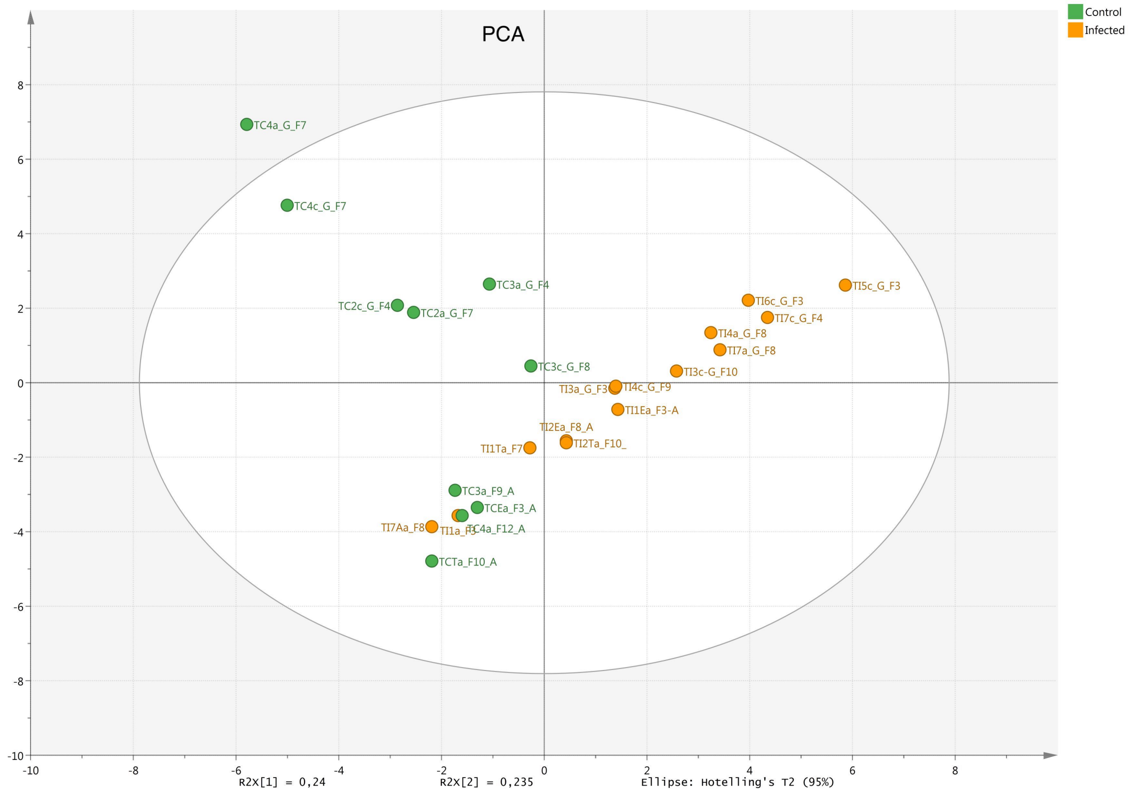

Figure S1: Principal Component Analysis of total volatile organic compounds, explaining 48% of the variability. Green circles represent healthy samples and yellow circles represent infected samples. A separation across the first principal component is clear.

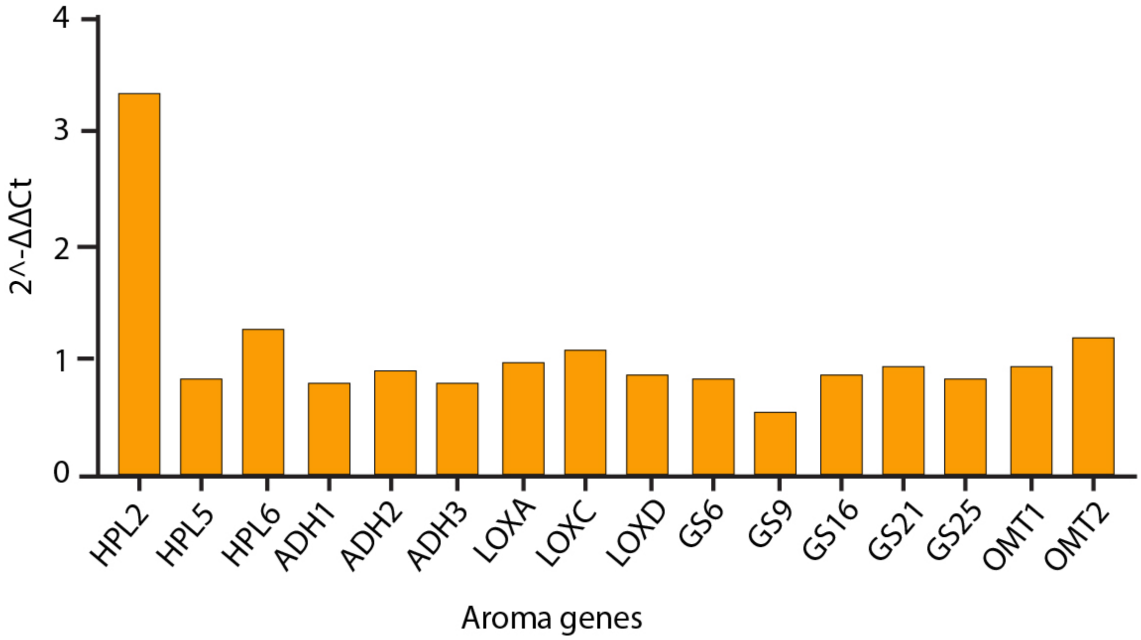

Figure S2: Plot with $2^{-\Delta\Delta C_T}$ values between control and infected samples for all the genes tested. Genes with value below 1 were not considered for the further experiments. The expression of certain genes (Fig. 6) was further determined using a standard curve, as described in Materials & Methods.

Table S1: Free and glycosidic bound volatile organic compounds expressed as percentage of total volatile organic compounds content, in both control and *Botrytis cinerea* infected Trincadeira grapes. Free and glycosidic form volatile organic compounds were analysed in separate runs.

**VOCs**	**RI**	**Free form percentage**		**Glycosidic bound percentage**	
		Control	Infected	Control	Infected
Ethanol	493	7.0	37.4	41.8	82.2
Isopropyl Alcohol	495	7.0	9.9		t
Acetaldehyde	497	24.0	5.1		
Ethyl acetate	603		17.5	t	9.7
Acetic acid	606	t	t	t	0.6
Isoamyl alcohol	722		0.8	5.2	1.7
Amyl alcohol	839				0.2
Hexanal	840	6.0	0.4		t
2-*trans*-Hexenal	866	32.5	7.2		
cis-2-Hexen-1-ol	882	2.3	6.9		
n-Hexanol	882	15.6	11.3	9.2	0.5
Isoamyl acetate	882				0.6
2-Methyl butyl acetate	882		t	t	0.3
Hexanoic acid	970				t
Ethyl hexanoate	965			0.5	0.1
Hexyl acetate	995		t	t	0.3
2-Phenylethanol	1064	t	0.2	0.2	0.3
n-Nonanol	1148		0.1	0.5	0.3
Octanoic acid	1149				t
Ethyl octanoate	1177		t	2.4	0.1
2-Phenylethyl acetate	1222		0.2	t	0.3
Ethyl nonanoate	1273			1.1	t
Nonyl acetate	1300			t	0.2
Decanoic acid	1356			t	0.2
Hexyl hexanoate	1375			0.5	
Ethyl decanoate	1387			18.7	0.9
Dodecanoic Acid	1550				0.1
Ethyl dodecanoate	1580			15.2	0.6
Ethyl tetradecanoate	1774			1.2	0.1
n-Octadecane (C18)	1800	1.2	1.1		
n-Nonadecane (C19)	1900	2.7	1.6		
Ethyl hexadecanoate	1936			2.4	0.4
n-Eicosane (C20)	2000	1.5	0.1		
n-Heneicosane (C21)	2100	t	0.1		
Linoleic acid	2137			1.1	0.3
n-Docosane (C22)	2200	t	t		

RI: Retention index relative to C_4-C_{22} *n*-alkanes on the DB-1 column. t: traces (<0.05%)

Table S2: List of primers selected from the literature that were used for the qPCR analysis. Tm: Melting temperature in ºC.

Gene ID	Protein	Orientation	Sequence	Tm(ºC)
GSVIVP00014710001	HPL2	Forward	GAGAGGAAGCTTGCCACAAC	58
		Reverse	AGACTTCATCAGCGGCATCT	
GSVIVP00036456001	HPL5	Forward	CTTCTTCCTCTCTTCCCCTCA	58
		Reverse	AGAACTGGTCACGGCCTTC	
GSVIVP00036457001	HPL6	Forward	CGAGGCAGACTTCAATGACA	58
		Reverse	TTGACGGTAAGGGAAAGGTG	
GSVIVP00014303001	ADH1	Forward	GGTCAAGTCATCTGCTGCAA	58
		Reverse	CGAAAATTCGAGGGAACAAA	
GSVIVT01026510001	ADH2	Forward	GCGTTGAGTGTACCGGAAAT	58
		Reverse	TTTCCACCACTGAAGGAAGG	
GSVIVP00014300001	ADH3	Forward	AGAGGACTCTCAAGGGCACA	58
		Reverse	TCCCCCTTCAGCATGTAGTC	
GSVIVT00024672001	LOXA	Forward	GCAAATCAAAGGGACAACGCTGTATGG	58
		Reverse	TGCTTCCACTGCGGCTTCC	
GSVIVT00022801001	LOXC	Forward	TGGTGGAAGGAAGTCAGGGAAGAG	58
		Reverse	TGGGCGGTTTGGGAGGTAGC	
GSVIVT00013309001	LOXD	Forward	ACCCACCAAATCGTCCCACACTATG	58
		Reverse	ACCTCTTCGTTGTCTGTCCACTCTG	
GSVIVT01030549001	OMT1	Forward	CTCCGCATAGCCGATATCAT	58
		Reverse	GAGAGTTTCTCCGCCATCTG	
GSVIVT01030545001	OMT2	Forward	ACAAAGGAGTGTCCCACGTC	58
		Reverse	GGCTCATAGCCATCTTCTCG	
GSVIVT00006898001	GS6	Forward	ACCCTTCAGCAACTCCACAG	58
		Reverse	TCCAGGTTCACACAAGGAGA	
GSVIVT01032025001	GS9	Forward	GGAGTGGGGAACCTATTCCT	58
		Reverse	CATGTCCTCAATTCGTGCAT	
GSVIVT00022347001	GS16	Forward	CAAGGGCAAACTCTTTTTGG	58
		Reverse	ATTGACCATGCGAAGTACCC	
GSVIVT01034947001	GS21	Forward	TCTTCAATGGAAGCTGCTGA	58
		Reverse	CTATTCCCATCCGGAACACA	
GSVIVT01014400001	GS25	Forward	AATATTGCCTGGGTGTTGGA	58
		Reverse	TTGGAACCCCATCTCTTTCTC	
VIT_04s0044g00580	Actin	Forward	GGTCAACCATGTTCCCTGGTATT	58
		Reverse	GGAGCAAGAGCAGTGATTTCCTT	
VIT_06s0004g03220	EF1α	Forward	CGTCATAGTTTTCTGCCTTCTTCC	58
		Reverse	TGCCACCGCCTATCAAGC	
